# HARP tracking using locally-uniform deformation assumption

**DOI:** 10.1186/1532-429X-16-S1-P370

**Published:** 2014-01-16

**Authors:** Ahmed S Fahmy, Safaa M ElDeeb, Ayman M Khalifa

**Affiliations:** 1 Biomedical Engineering, Cairo University, Cairo, Egypt; 2 Center for Informatics Science, Nile University, Cairo, Egypt; 3 Biomedical Engineering, Helwan University, Cairo, Egypt

## Background

Tracking of the myocardium points in tagged MRI images using the Harmonic Phase (HARP) method is widely used to estimate the myocardium regional function. Nevertheless, mistracking might occur in HARP due to errors in selecting the phase extraction filter or due to violation of phase-constancy near the myocardium edges [1]. In this work, we add a constraint that within any small neighborhood the myocardium deformation is uniform which allows using common methods of optical flow tracking such as the Lucas-Kanade algorithm [2]. We show that this assumption significantly improves the reliability of the HARP tracking especially at the borders of the myocardium.

## Methods

Given two tagged MRI images (with horizontal and vertical tags) acquired at time, t, the phase image is extracted using the HARP method in both images. Let these two HARP images be H1(x,y;t) and H2(x,y;t). Assuming that the myocardium deformation is uniform within a small neighborhood containing N pixels around a given pixel (x,y), a modified version of the LK optical flow tracking is used to track the pixel (x,y) between the two consecutive timeframes. The modification is based on calculating the image temporal and spatial gradients from both the horizontal and vertical HARP images. Numerical phantom of the heart has been used to determine the performance of the proposed method relative to the HARP at different noise levels. The error was recorded based on the ground truth values specified in the simulation. In addition, preliminary results on real datasets from 3 patients have been used to compare the two techniques, where points lying on myocardial contours (endo-, mid-wall, and epi-) have been tracked and the number of (visually) mistracked points was used as an indication of the reliability of the technique.

## Results

**Figure 1:**
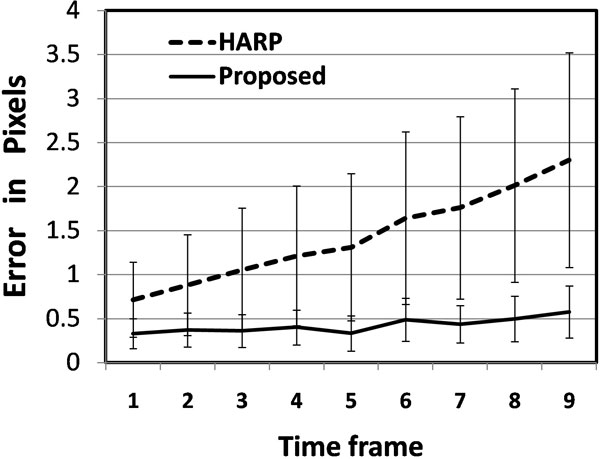
**Tracking error of the HARP and proposed method at the different time frames (numerical simulation)**.

**Figure 2:**
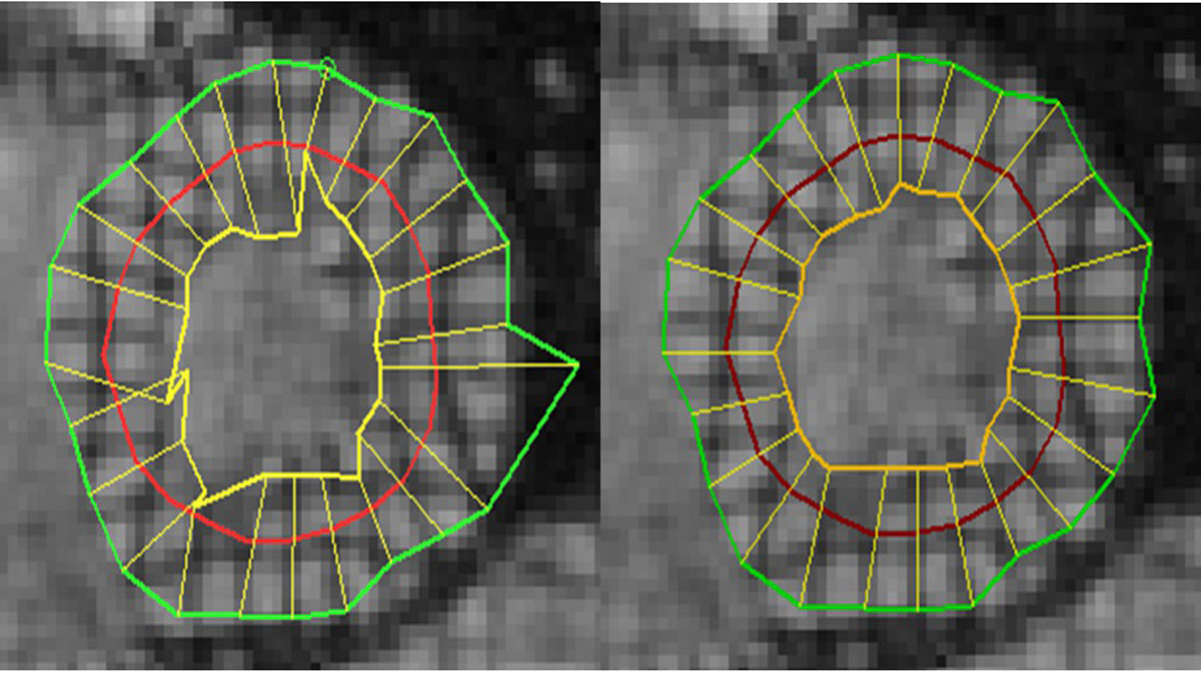
**Myocardium contours tracked using HARP (left) and proposed method (right)**.

Quantitative error analysis shows that the proposed method is much more accurate than the generic HARP tracking. As shown in Figure 1, at 0dB SNR,the mean error of the proposed method is increases from 0.25 to 0.5 pixels between the first and last timeframes with standard deviation increases from 0.15 to 0.35. On the other hand, the HARP mean error increased from 0.75 to 2.25 pixels with standard deviation 0.5 to 1.25. The qualitative assessment showed that both methods have comparable results at the midwall but the proposed method is much better (in terms of number of mistracked points) than the HARP at the endo- and epi-cardium contours. Figure 2 shows the myocardium contours at end systole tracked using HARP and the proposed method. It is clear from the figure that the mistracking of the HARP method is much more severe than that of the proposed method.

## Conclusions

A modified HARP method for tracking the myocardium in tagged MRI images has been proposed. The method has significantly improved the tracking accuracy at the edges of the myocardium.

## Funding

This work is fully funded by a grant from the ITAC program, ITIDA agency, Ministry of CIT, Egypt.
